# Comparative genomics reveals a constant rate of origination and convergent acquisition of functional retrogenes in *Drosophila*

**DOI:** 10.1186/gb-2007-8-1-r11

**Published:** 2007-01-18

**Authors:** Yongsheng Bai, Claudio Casola, Cédric Feschotte, Esther Betrán

**Affiliations:** 1 Department of Biology, University of Texas at Arlington, Arlington, TX 76019, USA

## Abstract

**Background:**

Processed copies of genes (retrogenes) are duplicate genes that originated through the reverse-transcription of a host transcript and insertion in the genome. This type of gene duplication, as any other, could be a source of new genes and functions. Using whole genome sequence data for 12 *Drosophila *species, we dated the origin of 94 retroposition events that gave rise to candidate functional genes in *D. melanogaster*.

**Results:**

Based on this analysis, we infer that functional retrogenes have emerged at a fairly constant rate of 0.5 genes per million years per lineage over the last approximately 63 million years of *Drosophila *evolution. The number of functional retrogenes and the rate at which they are recruited in the *D. melanogaster *lineage are of the same order of magnitude as those estimated in the human lineage, despite the higher deletion bias in the *Drosophila *genome. However, unlike primates, the rate of retroposition in *Drosophila *seems to be fairly constant and no burst of retroposition can be inferred from our analyses. In addition, our data also support an important role for retrogenes as a source of lineage-specific male functions, in agreement with previous hypotheses. Finally, we identified three cases of functional retrogenes in *D. melanogaster *that have been independently retroposed and recruited in parallel as new genes in other *Drosophila *lineages.

**Conclusion:**

Together, these results indicate that retroposition is a persistent mechanism and a recurrent pathway for the emergence of new genes in *Drosophila*.

## Abstract 2

Genome comparisons between 12 *Drosophila *species elucidate the origins of retroposition events that have led to the emergence of candidate functional genes.

## Background

Retrogenes are processed copies of genes that originate through reverse-transcription of a parental mRNA and insertion into the organism's genome [1]. This duplication mechanism produces a copy of the parental gene that should not contain introns, or most *cis*-regulatory regions. Processed copies of protein coding genes were described early in mammals because of their abundance. Retroposed gene copies are often believed to be pseudogenes because they lack regulatory regions and, as a consequence, they will often degenerate [2]. However, many of them are known to produce functional proteins and give rise to lineage-specific new functional genes [3-5].

Functional processed copies of genes can emerge as intronless duplications of the parental transcript [3,6] or recruit additional exons from the insertion site, producing a chimeric gene. The first retrogene described in *Drosophila*, *jingwei*, is of the latter type [5]. Even when processed copies of genes lose protein coding capacity, they can lead to regulatory RNAs (that is, micro RNAs [7]) or retroposed regulatory sequences [8]. Many will degenerate, becoming disabled non-expressed copies of genes, or be deleted from the genome. In humans, non-functional processed copies of genes (retropseudogenes) are found in large numbers (approximately 8,000) [4,9]. In contrast, dysfunctional relics of retroposed gene copies are relatively scarce in fruit flies (about 20 are detectable in *Drosophila melanogaster*) [10]. This contrasting pattern has been proposed as additional evidence in favor of the differences in deletion rate of nonfunctional DNA between these two species [10,11].

However, at what rate different genomes recruit new functional protein-coding genes from retroposed gene copies is still an open question. Recently, it has been estimated that human functional retrogenes originated at a rate of approximately one gene per million years (My) per lineage [12]. The rates at which functional retrogenes arose in other species are unknown. Here, we focus on functional protein coding retrogenes in *Drosophila*. We increased the list of known retrogenes in *D. melanogster *by identifying retrogenes independently of the location of the parental gene and at a less stringent level of protein identity (50%) than in previous studies (for example, [13]). This allows for the analyses of a most confident and comprehensive set of candidate functional retrogenes that originated in the lineage leading to *D. melanogaster*. The systematic assessment of the presence or absence of these retrogenes in the other 11 sequenced genomes of *Drosophila *provides a solid framework for inferring the age of each retrogene independently of sequence divergence analysis and for the calculation of a minimum rate of generation of functional retrogenes. We infer that functional retrogenes arose at a fairly constant rate of 0.51 genes per My per lineage. Many of these new genes recruited male germline function, suggesting an important role for retrogenes as a way of generating lineage-specific male functions. Unexpectedly, we show that three of the parental genes that gave rise to functional retrogenes described in *D. melanogaster *(*Cervantes*, *Dntf-2 *and *Ran*) have also independently given rise to functional retrogenes in parallel in other *Drosophila *lineages.

## Results and discussion

### Retrogene annotation

Additional data file 1 [including references [13-15]] shows the 97 candidate functional retrogenes that were identified in this study. These are annotated genes whose gene structure, compared to their closest paralogous gene in the genome (hereafter referred to as the parental gene), revealed that they most likely originated through retroposition. All these genes are well-supported functional genes; they are known genes (they have been named because their function is known), transcribed and/or display clear signals of purifying selection when compared to their parental gene (see Materials and methods and Additional data file 1). Out of the 97 retrogenes, we detected 3 events of tandem duplication that must have occurred after retroposition (Additional data file 1), which yield a minimum estimate of 94 retroposition events. This is a minimum estimate because: we used a cut-off level of protein identity of 50%, over at least 70% of the protein of both genes; we did not include partially processed copies, that is, copies that retained some introns but show the other features of retroposition [16]; and we cannot infer retroposition for intronless genes (which account for 18% of the annotated genes in the *D. melanogaster *genome) because there is no intron loss that can be used as the hallmark of retroposition.

Six of the retrogenes appear to have arisen from partial retroposition events, where, in the alignment of the protein coding region of parental and retrogene, the retrogene appears slightly shorter at the 5' ends compared to the parental gene. This type of truncation is typical of non-long terminal repeat retotransposons and can be attributed to incomplete reverse transcription, which initiates at the 3' end of the retroposon transcript [17]. The occurrence of 5' truncation in some *Drosophila *retrogenes suggests that, as in mammals, they are generated through the illegitimate action of retroposons' enzymatic machinery on cellular mRNAs [18]. There are also 11 retrogenes in which 5' or 3' introns in the untranslated region (UTR) or coding regions were acquired from the region of insertion (Additional data file 1). None of these are chimeric with other known genes; the new introns and exons represent newly recruited regions from previously non-coding intergenic DNA flanking the insertion site. There are already known examples of this [19,20]. In our annotation procedure, we required the pairs parental/retrogene to align over at least 70% of the proteins encoded by each gene and this precluded us from finding other types of chimeric retrogenes.

### Rate of origination of retrogenes

**Figure 1:**
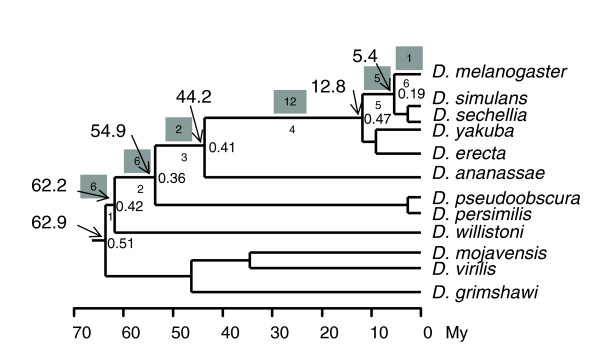
Retrogene phylogenetic distribution with respect to the analyzed sequenced genomes. Divergence times in each node are indicated. Branches are numbered below and the number of retroposition events in the particular internal branches is shown on top of the branch. The estimated rate of retroposition is shown in every internal node.

The presence/absence of a particular retroposed gene in related species can help in dating the retroposition event in a phylogenetic context. Together with reliable estimates of species divergence times [21], these data can provide a robust estimate of the rate of gene acquisition by the genomes. Here we used the wealth of data from 11 recently sequenced *Drosophila *genomes to date the origination of each retrogene found in *D. melanogaster *over the last approximately 63 My of *Drosophila *evolution (see Materials and methods). Figure 1 summarizes our findings for the retrogenes, while phylogenetic inferences for parental genes and tandem duplication events of retrogenes and the details of all inferences (that is, presence/absence in the different genomes) are given in Additional data file 2. We have studied the correlation between our gene age estimate and either K_S _or K_A _(Additional data file 3); we observed a significant correlation for these (Kendall's τ_Ks _= 0.6716, *P *< 0.000001; and Kendall's τ_Ka _= 0.4712, *P *= 0.00015) [22]. At the same time, we observed that variance increases with gene age for both K_S _and K_A _(Additional data file 3). This shows that our age estimates are congruent with the sequence divergence of the retrogenes from their parental genes, but that sequence divergence may not be a completely satisfactory measure of age.

From these data, a minimum rate of retrogene origination of 0.51 (32 genes/62.9 My) genes per My per lineage can be estimated (Figure 1). This means that approximately one functional retrogene was generated every 2 My during the *Drosophila *radiation. Many studies suggest that there is a high rate of generation of processed copies of genes in mammals according to the large number of processed copies of genes found in the genomes [4,23]. This has been related to the preponderance and high level of activity of L1 retrotransposable elements, which are able to provide the enzymatic machinery for the retroposition of cellular genes [18]. However, independently of the rate of generation of processed copies of genes in mammals, the rate at which new functional retrogenes arose in this lineage was projected to be much lower because the vast majority of retrogenes are obvious pseudogenes [4,23].

Recently, the rate of functional retrogene origination in primates during the last approximately 63 My of evolution (roughly the same timeframe examined in the present study) was estimated to be 1 retrogene per My per lineage [12]. Our results indicate, therefore, that the rate of functional (coding) retrogene acquisition is likely of the same order of magnitude in *Drosophila *and primates. It should be noted that the rate estimated by Marques *et al*. [12] for human retrogenes relies on synonymous divergence to assign genes to a particular lineage, a method that may not be completely accurate for dating retroposition events (Additional data file 3). In contrast, our method was independent of sequence divergence calculations and the molecular clock and, thus, may be viewed as more reliable. On the other hand, the comparison of the amount of constraint genes versus pseudogenes used by Marques *et al*. [12] to assign functionality of retrogenes is a very stringent approach that may lead to a severe underestimate of the actual rate of new genes originating by retroposition in the primate lineage.

To explore if the rate of retrogene origination has been constant throughout the period examined, we estimated the rate in every internal node of the *Drosophila *phylogeny (Figure 1). These estimates, around 0.45 (ranging from 0.51 to 0.36), are very similar for every node except for the youngest internal node (0.19). However, this estimate is based on a very small sample size (only one functional retrogene was gained during this period). We also have to consider that divergence times are accompanied by considerable standard errors [21]. Nonetheless, the results strongly suggest that the rate of functional retrogene origination has been fairly constant during the last approximately 63 My of the *Drosophila *radiation and no burst of retroposition can be inferred from this data, unlike those observed in primates [12].

### Retrogene origination pattern

Similarly to Betrán *et al*. [13], we tested whether functional retrogenes were produced in excess from X chromosome parental genes and transposed to autosomal locations. Consistent with previous results, we found a very significant excess of autosomal retrogenes originating from parental genes located on the X chromosome in *D. melanogaster *(*P *= 0.000001; Additional data file 5). Other studies suggest that this is consistent with mammals [4].

Several hypotheses have been put forward to explain this pattern of duplication. It is known that X chromosomes experience inactivation in males during spermatogenesis [24,25]. Thus, a mutant with a newly retroposed gene in an autosome might have some advantage over the ancestral individual because it can carry out a function required in male germline cells after X chromosome inactivation [4,6,13,24]. Recently, it also has been suggested that, according to sexual antagonism models, the autosomes can be a more favorable location for male-biased genes [26-28].

### Gene ontology categories represented in the parental/retrogene pairs

We examined the Gene Ontology (GO) categories represented in our parental/retrogene pairs ([29]). The range of functions is very diverse and eight parental/retrogene pairs have no known function. We found some interesting GO categories represented in our pairs. Many of these are related to male-specific function during spermatogenesis.

We found four parental/retrogene pairs that are proteasome component proteins: *Pros29*/*Prosα3T*, *Pros35*/*Prosα6T*, *Pros28.1*/*Pros28.1A *and *Prosβ5R1*/*CG31742*. Three of the retrogenes (*Prosα3T*, *Prosα6T *and *Pros28.1A*) are transcribed only during late spermatogenesis, while the parental genes are widely expressed [30,31]. All of these retrogenes are located on autosomes and one of them originated from a parental gene located on the X chromosome. This gene (*Pros28.1A*) thus fits the expectation of the out of X/male function pattern [13]. Other additional proteasome component proteins have been shown to be male-specific and it has been suggested that a sperm-specific proteasome is assembled and has a function different from the housekeeping proteasome in testis [31]. Our results demonstrate that three of the testes-specific proteasome components are, in fact, retrogenes that originated from housekeeping genes and subsequently recruited their male-specific function. Interestingly, these three retrogenes are not present in all the species of *Drosophila *examined in this study. *Prosα6T *originated in branch 1 (Figure 1) and it is present in only nine of the sequenced genomes. *Prosα3T *and *Pros28.1A *originated in branch 4 and they are present in only five of the sequenced genomes. Therefore, it is likely that the alleged testes-specific proteasome is of fairly recent origin and it is even tempting to speculate that its emergence contributed to species boundaries (that is, hybrid sterility). However, there are no mutants for these genes and, therefore, it is not known if the lack of their function can cause sterility or any other effect.

We also found two retrogenes predicted to encode ribosomal proteins (*RpS15Ab *and *RpL37b*), which are both autosomal retrogenes derived from X-linked ribosomal parental genes. Interestingly, Vinckenbosch *et al*. [32] also identified two functional retroposed copies of ribosomal proteins in humans that were also derived from X-linked genes transposed to autosomal locations. Together, these findings provide further support for the out of X hypothesis and are contrary to the belief that duplicates of ribosomal proteins are generally lost because of dosage effects [32]. Many other active ribosomal protein retrogenes, and even more inactive ones, have been found in mammals [33].

In addition, our retrogene set includes 15 retrogenes with similarity to known mitochondrial gene functions: *CG17856*, *CG6255*, *CG4706*, *CG9582*, *tomboy40*, *Hsp60B*, *CG9920*, *EfTuM*, *CG14508*, *CG5718, CG11913*, *CG10748*, *CG10749*, *CG18418 *and *CG7514*. Many of the retrogenes in these pairs (87%) are expressed in testis and some of them are known to have testis-specific functions. In testes, mitochondria are known to change shape (condense) and change function during spermatogenesis [34]. While spermatogonia can utilize aerobic pathways (that is, glucose) for energy production, spermatocytes have limited access to glucose and rely on lactate and pyruvate from Sertoli cells. These changes are accompanied by changes in gene expression [34]. Some of these changes may be accomplished through gene duplication followed by evolution of a male-specific pattern of expression for one of the paralogs. Our results suggest that retroposition is a major mechanism underlying the genetic innovation necessary for this physiological transition.

In *Drosophila*, testis-specific mitochondrial outer membrane translocators (*tom *genes) have been described (*tomboy20 *and *tomboy40*) [35]. These are duplicates of *tom20 *and *tom40 *[35]. Both *tomboy20 *and *tomboy40 *are male-specific intronless genes with a closely related intron-containing gene homolog, suggesting that they were generated by retroposition. However, only the pair *tomboy40*/*tom40 *was retrieved by our search. The relatively low identity (47%) between Tomboy20 and Tom20 proteins [35] explains why the other pair was not included in our set. *Tomboy20*/*tom20 *is an autosome to autosome retroposition, while *tomboy40*/*tom40 *was an X to autosome event, in accord with the prediction of the out of the X hypothesis. The exact functions of *tomboy20 *and *tomboy40 *are not known, but it is plausible that these proteins are incorporated into an outer membrane translocation complex that has mitochondrial male specificity for certain male proteins [35]. Another example of a male-specific gene related to mitochondrial function in *Drosophila*, identified by our screen as a functional retrogene, is *hsp60B*. It encodes a heat shock protein that has been reported to be essential for spermatid individualization [36].

Additional examples of retrogenes that are testis- and/or male-specific in our set include *Arp53D*, an actin related protein expressed only in testis, *gskt*, a male germline-specific protein kinase required for male fertility and recently named *mojoless *(R Kalamegham, D Sturgill, E Siegfried, and B Oliver, personal communication), *roc1b*, which causes male sterility, *CG9573*, which maps in a male sterility locus [29], and *Dntf-2r*, which is highly expressed in testis [3]. Therefore, many retrogenes appear to have evolved male functions (see also the 'Out of the testes' section).

### Out of the testes

Recently, a very forward hypothesis has been suggested after studying the pattern of expression of likely functional retrocopies in primates: the 'out of the testes' hypothesis [32]. This hypothesis states that functional retrogenes are initially expressed in testes, which may contribute to their immediate preservation, but later acquire a higher and broader tissue expression, which may eventually lead to the evolution of other new functions.

Tissue expression analyses revealed that a higher percentage of parental genes than retrogenes are represented in all the libraries analyzed, with the exception of an adult testis library (AT; Additional data file 5, adult testis 2). However, the percentage of retrogenes that are expressed in adult testis is 53%, while 42% of the parental genes are expressed in this tissue. Parental genes also tend to be expressed in more tissues than retrogenes do: average number of libraries (that is, tissues) is 7.15 versus 2.70 for the retrogenes. This reveals that many retrogenes are expressed primarily in testis, similar to the observations in human [32].

To compare our results with the human results we studied whether young retrogenes are expressed at lower levels than older ones [32]. We did not find a significant positive correlation between the number of hits for expressed sequence tags (ESTs)/cDNAs in the libraries and K_A _or K_S _(Kendall's τ_Ka _= -0.2530, *P *= 0.0020; Kendall's τ_Ks _= -0.0546, *P *= 0.5042; note that one correlation is significant but negative). We also tested against our estimate of the age of the retrogene. We consider that the age of a retrogene is the middle point in the lineage in which it originated or 62.9 My if it is present in all the species. There was again no significant correlation between age of the retrogenes and the expression level (Kendall's τ = 0.1396, *P *= 0.0874), contrary to the observations in humans.

We directly studied the relationship between expression level in testis (that is, number of hits in the AT library) and age given by K_A_, K_S _and phylogenetic distribution to address the 'out of the testes' hypothesis. None of these relationships were significant: Kendall's τ_Ka _= 0.0065, *P *= 0.9482; Kendall's τ_Ks _= -0.0416, *P *= 0.6763; and Kendall's τ = 0.0398, *P *= 0.6902. Finally, we also explored whether the proportion of testis EST hits decreases with age of the retrogene, as had been observed in humans [32]. We did not find a significant positive correlation between this proportion and the measures of age: Kendall's τ_Ka _= 0.0820, *P *= 0.3153; Kendall's τ_Ks _= 0.1564, *P *= 0.0555; and Kendall's τ = 0.0272, *P *= 0.7390.

In sum, the results of these analyses do not concur with any of the predictions made by the 'out of the testes' hypothesis [32]. Many *Drosophila *retrogenes are expressed primarily in testes but we did not see a pattern in which younger genes are expressed in testis and older genes expressed in more tissues than testis and at a relatively lower level in this tissue.

### Chimeric retrogenes

In this work, we consider a gene to be a chimeric gene if it recruited additional introns and exons from the genomic regions flanking the insertion site. Out of eleven such chimeric retrogenes identified (Additional data file 1), one recruited a new coding region, eight recruited 5' UTR additional introns and exons, and two recruited 3' UTR additional introns and exons. It is important to point out that, in all the cases, the new introns and exons were recruited from non-coding regions flanking the insertion site and apparently not from pre-existing genes. However, the design of our screen to identify retrogenes somewhat precluded finding chimeric retrogenes that originated from two existing genes (see Materials and methods).

These data provide compelling cases of intron gains in multiple genes. But how long does it take to acquire a new intron? We investigated whether those genes that acquired new intron/exon structures are older than average. We again used K_A_, K_S _and phylogenetic distribution as measures of age and compared only retrogenes for which UTRs have been annotated [29]. The average K_A _and K_S _(± standard error) of the chimeric retrogenes were 0.2241 ± 0.0440 and 6.8977 ± 1.6752, respectively. The average K_A _and K_S _of retrogenes for which UTRs have been annotated were 0.2584 ± 0.0210 and 10.2259 ± 1.0045. According to the phylogenetic distribution (Figure 1), the average age estimated as described above is 59.7727 ± 2.9817 My in chimeric genes and 56.2100 ± 2.4106 My in other retrogenes for which UTRs have been annotated. From these data we conclude that chimeric retrogenes do not appear to be older than other retrogenes and, therefore, retrogenes do not need extra time to acquire new introns and exons from the region of insertion. In fact, these acquisitions most likely occur rapidly after the duplication event, as has been observed for other chimeric genes that arose from retroposition [5].

### Evidence of retrogenes subsequently relocated

While assessing the presence/absence of a particular gene in the different sequenced genomes, we observed three instances where a retrogene had apparently been relocated from its initial insertion site. In these cases, a clear ortholog of the retrogene could be identified in a given genome, but it was not in a syntenic location compared to other related genomes (see Materials and methods and Additional data file 6). We consider as evidence of clear orthology the fact that, in these gene families, parental and derived genes are the best hit in the genomes and, in the phylogeny, they group completely apart, as expected when the duplication is ancient, independently of their location in a particular genome (Additional data file 6). *CG7423 *and *CG9013 *seem to be in a different Muller chromosomal arm in *D. virilis, D. mojavensis *and *D. grimshawi*. *CG6036 *is on another Muller chromosomal arm in *D. pseudoobscura *and *D. persimilis*. We consider these events the product of duplication and loss of the gene in the original position and assume that the gene is as old as the relocated gene in our gene age analysis. These events could contribute to further diversify the functionality of retrogenes and have a direct impact on the evolutionary trajectory of the species, leading, in some instances, to hybrid breakdown [37].

We consider the hypothesis of independent insertion less parsimonious given the way genes group in the K_S _and protein tree (Additional data file 6), that is, all parental and all retrogenes group apart, but if the mutation rate is higher in the retrogene location, we could be seeing some type of long branch attraction in the case of *CG7423 *and *CG9013*. *CG6036 *is on another Muller chromosomal arm only in *D. pseudoobscura *and *D. persimilis *and that is more difficult to explain with independent insertions because it is an internal lineage.

Another possible, but again likely less parsimonious, explanation to the relocation of retrogenes in different lineages would be the existence of two ancient retroduplications and the loss of one or the other duplicate gene in the different lineages. For *CG7423 *and *CG9013*, a duplicate would be lost in the *Drosophila *subgenus and a different one in the *Sophophora *subgenus. In the case of *CG6036*, multiple independent losses of the same gene will have to occur in the *Drosophila *subgenus and the *Sophophora *subgenus.

### Recurrent and convergent recruitment of functional retrogenes during *Drosophila *evolution

We define as recurrent recruitment the situation whereby the same parental gene has given rise repeatedly to several functional retrogenes within the same lineage of *Drosophila *(that is, the closely related paralog of two intronless genes was the same parental gene). Three parental genes (*Cnx99A*, *CkIα *and *Vha16*) produced two, two and three retrogenes, respectively. For *Cnx99A *the two duplications are only present in *D. melanogaster*, *D. simulans*, *D. sechellia *and *D. mauritiana*. For *CkIα *the two duplications predate the *Drosophila *genus diversification. Two of the *Vha16 *duplications predate the *Drosophila *genus diversification. The other one is absent in the *Drosophila *subgenus. Why the number of functional genes has grown in these three gene families in the *D. melanogaster *lineage through retroposition is not understood. While examination of the number of retroposed nonfunctional copies of genes can provide evidence of what transcripts are more likely to be retroposed (that is, stable mRNAs or mRNAs encoding soluble proteins, the latter being transcripts that stay longer in the cytoplasm and do not get targeted to the endoplasmic reticulum [38]), only deeper understanding of the role of the functional copies can reveal why some are kept functional.

**Figure 2:**
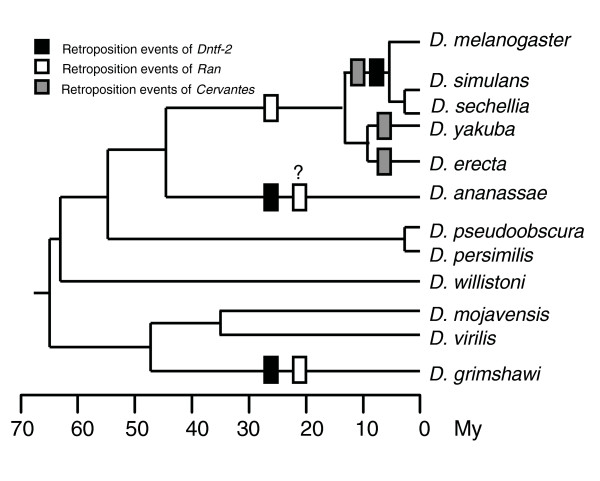
*Drosophila *phylogeny showing a summary of our inferences about the time of the independent parallel retroposition events from the parental genes *Cervantes*, *Dntf-2 *and *Ran*. Note that some of these events (for *Dntf-2 *and *Ran*) overlap, as discussed in the text.

We also explored our dataset for convergent recruitments of retrogenes in different lineages of *Drosophila *and found three instances. Two independent lines of evidence support these parallel recruitments: different chromosomal location and higher similarity to the parental gene than to other retrogenes in a K_S _tree (Additional data file 7). In one case, *Dntf-2 *seems to have given rise to retrogenes three times independently in different *Drosophila *lineages (Additional data file 7, Figure 2). It gave rise to the retrogene in our set that is present in four species (*D. melanogaster*, *D. simulans*, *D. mauritiana *and *D sechellia*) [3] on chromosomal arm 2L. It seems to have given rise to a retrogene in the *D. ananassae *lineage and another independent retrogene originated in the lineage of *D. grimshawi*. These two additional retrogenes map in the chromosomal arm homologous to 3L in *D. melanogaster*. In another case, *ran *seems to have given rise to retrogenes at least twice (but likely three times) in different lineages. It gave rise to the retrogene in our set that is present in all the species of the *D. melanogaster *subgroup (Figure 2, Additional data file 2) on chromosome 3L and appears to be evolving very fast even at synonymous sites (Additional data file 7; note the very long branches). *Ran *seems to have given rise to another possibly fast evolving retrogene in the *D. ananassae *lineage located in the chromosomal arm homologous to 2L of *D. melanogaster*. We believe these two events could be independent, despite the fact that they group together in the K_S _tree, because the grouping can be an artifact of both sets of genes evolving very fast even at synonymous sites, possibly due to their lack of codon bias or a higher mutation rate in their chromosomal location. Finally, a clear independent retrogene in the lineage of *D. grimshawi *seems to have originated from *ran *and is located in the chromosomal arm homologous to 3L of *D. melanogaster *(Figure 2). Seemingly intact and full-length open reading frames are evident for all these newly duplicated retrogenes, making all these likely functional copies (data not shown).

Remarkably, it has been previously established that the proteins encoded by *Dntf-2 *and *ran *interact with each other during the transport of proteins to the nucleus. Thus, the overlapping presence of duplicates of both genes, independently acquired by retroposition, in some lineages may have an adaptive meaning, in particular if they overlap in their expression. Interestingly, all three independent retropositon events involved retroposition from the X chromosome to an autosome location, which has been claimed to be positively selected for in the genome [4,13]. Interestingly, *Dntf-2r *is highly expressed in the male germline [3]. It is possible that multiple parallel retroposition events of *Dntf-2 *and *ran *took place and that they fixed in the population under positive selection due to the fact that they encode proteins that physically interact and function together in the same cellular processes in the male germline. We are currently investigating if the expression pattern of the *ran *retrogenes overlaps that of *Dntf-2 *retrogenes.

The third example of convergent retrogene recruitment corresponds to the retrogenes that originated from the parental gene *Cervantes *(*CG15645*) that we revealed in a recent study [39]. We named the retrogene in *D. melanogaster Quijote *(*CG13732*), and discovered that *Quijote *is present in only four species of *Drosophila *(*D. melanogaster*, *D. simulans*, *D. sechellia *and *D. mauritiana*). In that study, we also inferred that retroposed copies of *Cervantes *also originated in the lineages leading to *D. yakuba *and *D. erecta *and this occurred independently in the three instances (Additional data file 7, Figure 2). This is one example of parallel recruitment of a retrogene from the same parental gene in different *Drosophila *lineages. Here again, the convergent retroposition events involved retroposition from the X chromosome to an autosome, possibly revealing the selective pressure of X inactivation or sexual antagonism, as introduced above. In agreement with this 'out of the X convergent event' hypothesis, a *Utp14 *retrogene involved in pre-rRNA processing and ribosome assembly has likely been recruited independently for male-function in four distinct mammalian lineages [40,41]. These were also X to autosomes retroposition events. The authors argue that the independent recruitment supports the hypothesis that it is highly beneficial for males to gain autosomal copies of the *Utp14 *gene to compensate for the silencing of the X chromosome during male meiosis, as discussed above.

Again, a possible, but less parsimonious, explanation to the parallel recruitment of retrogenes in different lineages would be the existence of several ancient retroduplications and the loss of all or all except one retrogene in different lineages and the action of gene conversion between parental genes and retrogenes right after the losses occur.

## Conclusion

This work provides the most accurate estimate of the rate of functional retrogene recruitment published to date for any species lineage (0.51 retrogenes/My). This rate was fairly constant for approximately 63 My of *Drosophila *evolution and its value is of the same order of magnitude of the approximate rate recently published for the human lineage (1 retrogene/My) [12]. Many of the *Drosophila *retrogenes are expressed primarily in the male germline and have often evolved male-specific functions. In addition, a very interesting pattern is revealed from our searches of convergent recruitments of retrogenes in different lineages. Three prolific parental genes (*Dntf-2*, *ran *and *cervantes*) seem to have produced retrogenes in parallel in different lineages (Figure 2). All of these events fit the preferential X chromosome to autosome traffic of retrogenes [13]. It is likely, therefore, that positive selection has repeatedly driven the export of functions from the X chromosome to autosomes. We are now studying in more detail the molecular evolution and pattern of transcription of the convergently acquired retrogenes to test this hypothesis.

## Materials and methods

### Retrogene annotation

We conducted this analysis by surveying the whole *D. melanogaster *genome in the Ensembl dataset (version 36) for retrogenes using similar computational approaches to those previously described [4,13], with a few modifications. The FASTA34 package [42] was used to perform similarity searches with each single peptide in the Ensembl dataset against all other peptides to identify duplicate genes. We lowered the level of amino acid identity between protein pairs to 50% and the overlap level between two proteins to 70%. The parental gene was assigned to the highest amino acid identity hit. To be called a retrogene, we required that the region of similarity between an intronless gene and a parental gene spans all the introns of the parental gene coding region. However, we also looked at genes with small numbers of introns (<4) and additionally identified 11 parental/retrogene pairs with ≥33% difference in the number of introns; these are the cases where the retrogene recruited new introns. Partial retrogenes were also noted (six cases); these are intronless genes that do not span all the introns of the parental gene.

### Tissue expression analyses

We downloaded a *D. melanogaster *EST/cDNA database (October 2003 release) locally from the Berkeley *Drosophila *Genome Project [43]. We queried these data using Blastn [44] with our retrogene and parental gene dataset. Tissue expression was assessed using a similar approach to the one followed by Emerson *et al*. [4], except that here we lowered the nucleotide identity level to 97% because, in *Drosophila*, we expect a relatively higher level of intrapopulation polymorphism [45]. The total number of sequences in the EST/cDNA database for each of the 15 libraries we downloaded are as follows: AT (adult testis), 26,226; GM (ovary), 12,765; UT (adult testis), 1,368; EC (fat body of larvae), 10,460; EP (mix of embryo, imaginal disks, and head), 9,423; EN (mbn2 cell line), 8,068; LP (larvae and pupae), 17,204; HL (head), 3,506; SD (schneider cell culture), 23,150; CK (embryo endoplasmic reticulum), 1,673; GH (head (male + female)), 29,132; EK (mix of embryo, imaginal disks and head), 80,857; LD (embryo), 43,509; RH (head normalized), 58,393; RE (embryo normalized), 67,658. The tissue expression for a gene was obtained by averaging the tissue hits for all transcripts of that gene. This type of expression data allows for the assertion of expression of duplicate genes without the confusing effects of sequence similarity between duplicates [13].

### Revealing constraint: K_A_/K_S _calculation

As described by Betrán *et al*. [13], we used a K_A_/K_S _of 0.5 between a retrogene and a parental gene as the conservative cut-off value that reveals constraint in the retrogene lineage. The Codeml program PAML 3.1[46] was run twice for every gene pair; first to fix K_A_/K_S _= 0.5 and second to estimate the ratio, to test if K_A_/K_S _is significantly smaller than 0.5 using a likelihood ratio test.

### Checking of presence/absence of retrogenes and parental genes and their structure in other *Drosophila *genomes

We estimated the time of the retroposition events by checking the presence/absence of retrogenes and their parental genes in the 11 additional *Drosophila *species that have now been fully sequenced. We used three approaches to assign orthology between the genes under examination. First, we required that at least one of the two nearest gene neighbors be present on either side of the gene under scrutiny (conservation of synteny). This was done by looking at the translated similarity searches (tBLASTn [44]) against the assemblies of 11 related *Drosophila *species (Comparative Analysis Freeze 1 [47]). Sequence sources: *D. erecta*, *D. ananassae*, *D. mojavensis*, *D. virilis *and *D. grimshawi *were sequenced by Agencourt, Inc (Beverly, MA, USA); *D. simulans *and *D. yakuba *were sequenced by Washington University; *D. sechellia *and *D. persimilis *were sequenced by the Broad Institute; *D. willistoni *was sequenced by TIGR; *D. melanogaster *was sequenced by the Berkeley *Drosophila *Genome Project and Celera [48]; and *D. pseudoobscura *was sequenced by Baylor [49]. Because chromosomal rearrangements (that is, paracentric inversions) could potentially result in scrambling of the genes along a chromosomal arm [50], we reasoned that the conservation of microsynteny, as given by the two neighboring genes, might not be sufficient to infer orthology. To increase the accuracy of our orthology assignment, we complemented this approach by a phylogenetic analysis using all protein hits of the selected gene in the related species with expected gene structure and looking for clear phylogenetic support (proteins of retrogenes or parental genes of the different species grouped together with a good bootstrap support and following the known topology) to assign orthology. This approach allowed us to find relocated genes, that is, retroposed genes homologous by descent but subsequently relocated to another chromosomal position. Convergent recruitments were suspected whenever the phylogenetic inference supported several retrogenes having higher similarity to the parental genes of their lineages than to the other retrogenes. Additional support for convergent recruitment was obtained from the K_S _tree and chromosomal location of the retrogenes being different (Additional data file 7). Finally, we also checked the synteny conservation up to five neighboring genes on either side for each selected gene identifying their predicted orthologous genes in the UCSC browser [51] in particularly ambiguous cases.

Examining the presence or absence of a particular gene and its structure in related species from the tBLASTn and BLAT hits can help reveal false positives in our retrogene annotation (that is, recent intron gain by a parental gene or intron loss by a retrogene) or wrong assignment of parental gene (that is, a parental gene being younger than a retrogene). Our analyses of the phylogenetic distribution of parental genes and retrogenes (Additional data file 2, Figure 1) revealed that the phylogenetic distribution of the parental gene was always the same or wider than those of the cognate retrogene. However, we found one case in which the lack of an intron in the alleged retrogene could be explained by genomic duplication followed by intron gain in the parental gene rather than by retroposition, that is, orthologous sequences of the parental gene are intronless genes, and one case in which it could be explained by genomic duplication followed by intron loss in the alleged retrogene, that is, orthologous sequences of the retrogne are intron-containing genes. We discarded these two pairs from our final dataset listed in Additional data file 1.

## Additional data files

The following additional data are available with the online version of this paper. Additional data file 1 is a table listing the retrogenes and parental genes, their location, gene structure and sequence analyses. Additional data file 2 is a table that shows our inferences of the presence/absence of retrogenes and parental genes in every *Drosophila *genome. Additional data file 3 is a figure that shows the K_S _and K_A _correlation with our phylogenetic assignment (gene age estimate). Additional data file 4 is a table that shows the statistical analysis of duplication between chromosomes. Additional data file 5 is a figure that shows the proportions of parental genes and retrogenes expressed in every cDNA/EST library analyzed. Additional data file 6 is a figure that shows the phylogenetic evidence for the gene relocation events. A K_S _(Nei-Gojobori method) neighbor-joining tree of some members of the gene family is shown. Bootstrap values are shown in the nodes after 10,000 replications. MEGA [52] was used for this phylogenetic reconstruction. Chromosomal location was inferred from the location of flanking genes in *D. melanogaster *and is also given. Additional data file 7 is a figure that shows the phylogenetic evidence for the convergent recruitment events. A K_A _and K_S _(Nei-Gojobori method) neighbor-joining tree of some members of the gene family is shown. Bootstrap values are shown in the nodes after 10,000 replications. MEGA [52] was used for this phylogenetic reconstruction. Chromosomal location was inferred from the location of flanking genes in *D. melanogaster *and is also given.

## Supplementary Material

Additional data file 1 Retrogenes and parental genes, their location, gene structure and sequence analyses. Click here for file

Additional data file 2 Inferences of the presence/absence of retrogenes and parental genes in every *Drosophila *genome. Click here for file

Additional data file 3 K_S _and K_A _correlation with our phylogenetic assignment (gene age estimate). Click here for file

Additional data file 4 Statistical analysis of duplication between chromosomes. Click here for file

Additional data file 5 Proportions of parental genes and retrogenes expressed in every cDNA/EST library analyzed. Click here for file

Additional data file 6 A K_S _(Nei-Gojobori method) neighbor-joining tree of some members of the gene family is shown. Bootstrap values are shown in the nodes after 10,000 replications. MEGA [52] was used for this phylogenetic reconstruction. Chromosomal location was inferred from the location of flanking genes in *D. melanogaster *and is also given. Click here for file

Additional data file 7 A K_A _and K_S _(Nei-Gojobori method) neighbor-joining tree of some members of the gene family is shown. Bootstrap values are shown in the nodes after 10,000 replications. MEGA [52] was used for this phylogenetic reconstruction. Chromosomal location was inferred from the location of flanking genes in *D. melanogaster *and is also given. Click here for file
