# Supplementary material for: Comparative genomics reveals a constant rate of origination and convergent acquisition of functional retrogenes in Drosophila
Source: Genome Biol. 2007 Jan 18;8(1):R11. doi: 10.1186/gb-2007-8-1-r11 (PMC1839131; doi:10.1186/gb-2007-8-1-r11)
Supplement: Additional data file 1 — Retrogenes and parental genes, their location, gene structure and sequence analyses. [file gb-2007-8-1-r11-S1.pdf]

Additional file 1. List of retrogenes and parental genes, their location, gene structure and sequence analyses. Probability value (P-value) is the significance level for the test of K_A/K_S being smaller than 0.5 [13]. Plus/minus indicate if the gene showed any hit in the EST/cDNA libraries explored (see Materials and Methods). First 76 genes are typical retroposed genes, there are 11 retroposed genes that recruited new introns and exons from the insertion site (chimeric retrogenes), next 6 genes are partial retroposition events of which 2 of them also recruited new introns and exons, and the last 6 genes are three events of retroposition with subsequent tandem duplication. Out of a total of 97 gene pairs, we have support for 94 retroposition events because there are three events of retroposition with subsequent tandem duplication (see Results and Discussion).

**Retrogene**	**Exp.**	**Chr.**	**Intron #**	**Parental gene**	**Chr.**	**Intron #**	K_A	K_S	**Ka/Ks**	**P-value**
RpS15Ab	+	2R	0	*RpS15Aa*	X	2	0.0096	0.0592	0.1622	0.1506489
Dntf-2r	-	2L	0	*Dntf-2*	X	3	0.056	0.1637	0.3421	0.3441529
CG9573	+	2L	0	*CG14213*	X	5	0.1949	0.3985	0.4891	0.911577
CG4960	+	3R	0	*CG8331*	2R	3	0.1216	0.4234	0.2872	**0.0357177**
CG5265	+	3R	0	*CG1041*	3R	4	0.1646	1.4073	0.1170	**4.30E-34**
CG5755	+	2L	0	*CG8931*	X	3	0.2466	1.8397	0.1340	**2.68E-19**
Atg8b	+	3R	0	*Atg8a*	X	2	0.0944	2.1472	0.0440	**2.93E-14**
CG17856	-	3R	0	*CG3560*	X	2	0.0854	2.2071	0.0387	**6.58E-15**
CG6255	+	3R	0	*Scsα*	3L	3	0.278	2.3254	0.1195	**1.31E-14**
Pglym87	+	3R	0	*Pglym78*	3R	2	0.1486	2.816	0.0528	**1.01E-22**
Cdlc2	+	2L	0	*Ctp*	X	2	0.0045	3.1211	0.0014	**3.62E-27**
CG32669	+	X	0	*CG10444*	2R	1	0.3626	3.3908	0.1069	**5.01E-22**
Prosα3T	+	3R	0	*Pros29*	2R	2	0.2433	3.9175	0.0621	**1.31E-19**
Prosα6T	+	2L	0	*Pros35*	2L	2	0.303	4.8167	0.0629	**5.57E-15**
Roc1b	+	3L	0	*Roc1a*	X	2	0.1794	5.0622	0.0354	**1.12E-10**
CG4408	+	3R	0	*CG2915*	2R	1	0.3707	5.2069	0.0711	**3.95E-22**
Rpl37b	-	2R	0	*Rpl37a*	X	2	0.1795	5.2297	0.0343	**1.28E-11**
CG2528	+	2L	0	*CG5355*	2L	8	0.2253	5.7176	0.0394	**5.93E-64**

Sep5	+	2R	0	*Sep2*	3R	1	0.1711	6.2976	0.0272	**8.58E-48**
CG10931	-	2R	0	*Wds*	X	2	0.4112	6.7121	0.0613	**1.87E-18**
Grx-1	+	2R	0	*CG6852*	3L	2	0.229	6.9093	0.0331	**4.09E-13**
Vha36	+	2R	0	*CG8310*	X	3	0.0937	7.7579	0.0121	**1.03E-33**
CG4706	+	3R	0	*Acon*	2L	3	0.1275	8.2319	0.0155	**4.43E-113**
CG31477	-	3R	0	*Sun*	X	3	0.1569	8.7359	0.0180	**9.35E-08**
Try29F	+	2L	0	*CG31954*	2L	1	0.4971	8.8477	0.0562	**2.07E-09**
CG9582	+	2L	0	*CG5254*	X	1	0.3731	9.4592	0.0394	**1.38E-21**
Trxr2	+	3L	0	*Trxr1*	X	3	0.1783	9.9287	0.0180	**1.57E-46**
CG5150	+	3L	0	*CG10827*	3R	1	0.3421	11.127	0.0307	**3.61E-29**
CG4701	+	2L	0	*Nmd*	2L	1	0.2806	11.8395	0.0237	**6.90E-36**
CG8986	+	2R	0	*CG14534*	2L	2	0.4868	12.4663	0.0390	**8.31E-08**
Pp2B-14D	+	X	0	*CanA1*	3R	12	0.1666	13.1396	0.0127	**9.97E-62**
CG1950	+	X	0	*Uch-L3*	3L	1	0.2943	13.9208	0.0211	**1.13E-22**
CG1287	+	3R	0	*CG2076*	X	5	0.2603	13.932	0.0187	**1.14E-27**
Ran-like	+	3L	0	*Ran*	X	2	0.3081	14.1202	0.0218	**4.71E-17**
CG6036	+	3R	0	*CG1906*	3R	5	0.3819	14.2758	0.0267	**1.80E-21**
CG9150	+	2L	0	*CG8757*	3L	2	0.3745	14.3447	0.0261	**2.70E-12**
Robl22E	-	2L	0	*Robl*	2R	2	0.3441	14.6062	0.0236	**1.25E-06**
CG3162	+	2R	0	*U2af50*	X	4	0.3305	15.0332	0.0220	**7.77E-29**
CG32238	-	3L	0	*CG8918*	X	3	0.3687	15.0435	0.0245	**1.47E-30**
CG32087	+	3L	0	*CG32675*	X	4	0.451	15.3933	0.0293	**2.15E-22**
CG8602	+	3L	0	*CG12194*	2L	4	0.1965	15.4682	0.0127	**5.59E-49**
CG13078	-	2L	0	*CG13077*	2L	2	0.4135	15.7243	0.0263	**3.35E-13**
CG10839	+	2L	0	*CG8800*	2R	2	0.3509	15.9145	0.0220	**1.95E-11**
Tomboy40	+	2R	0	*Tom40*	X	3	0.1976	16.8322	0.0117	**1.04E-39**
CG6873	-	X	0	*Tsr*	2R	3	0.3601	17.3385	0.0208	**6.64E-12**
CG31202	-	3R	0	*α-Man-I*	X	5	0.4577	17.8128	0.0257	**3.01E-18**
CG9254	+	2L	0	*Genel(2)01810*	2L	4	0.2052	18.9579	0.0108	**3.93E-48**
CG17003	+	X	0	*CG3967*	3L	6	0.5544	19.14	0.0290	**5.14E-08**
CG7423	-	X	0	*CG31715*	2L	3	0.2586	19.5918	0.0132	**2.06E-18**

Gs1	+	2L	0	*Gs2*	X	4	0.2546	19.7477	0.0129	**2.94E-41**
CG18735	-	2R	0	*CG4386*	2R	1	0.3936	19.8871	0.0198	**2.30E-16**
CG7542	+	3L	0	*CG10472*	3L	2	0.5109	20.5512	0.0249	**2.39E-14**
CG1409	+	X	0	*Blp*	3R	2	0.4302	21.5155	0.0200	**5.80E-09**
CG12362	+	3L	0	*Ari-1*	X	6	0.2867	22.5285	0.0127	**6.96E-40**
Quijote	+	3L	0	*Cervantes*	X	1	0.1581	0.2848	0.5551	0.691571
CG1924	-	X	0	*Cnx99A*	3R	9	0.1312	0.3851	0.3407	**0.01130312**
CG8629	+	3L	0	*CG8628*	3L	1	0.0927	0.5247	0.1767	**0.01689293**
αTub84D	+	3R	0	*αTub84B*	3R	1	0.0028	0.6307	0.0044	**3.10E-55**
Act87E	+	3R	0	*Act57B*	2R	1	0.0054	0.6713	0.0080	**3.45E-45**
Pros28.1A	+	3R	0	*Pros28.1*	X	2	0.1476	1.6006	0.0922	**2.74E-17**
Hsc70-4	+	3R	0	*Hsc70-1*	3L	1	0.1029	12.1249	0.0085	**3.16E-107**
CG13069	+	3L	0	*CG13067*	3L	1	0.4373	12.4782	0.0350	**2.35E-05**
CG7094	+	2L	0	*CkIα*	X	3	0.3118	16.0636	0.0194	**1.31E-27**
CG10104	+	2R	0	*CathD*	2R	1	0.3512	18.311	0.0192	**2.97E-27**
CG7768	+	3L	0	*Cyp1*	X	1	0.0954	19.5718	0.0049	**1.62E-33**
CG9906	-	X	0	*Cnx99A*	3R	9	0.0897	0.3049	0.2942	**0.001216831**
CG7197	+	3L	0	*Arf79F*	3L	4	0.4104	5.049	0.0813	**5.56E-08**
CG9722	-	3R	0	*Nmda1*	2R	4	0.3759	8.612	0.0436	**1.02E-13**
CG9013	-	2R	0	*Vha16*	2R	3	0.2324	11.9124	0.0195	**8.12E-20**
Hsp60B	+	2L	0	*Hsp60*	X	2	0.2552	13.0582	0.0195	**1.43E-42**
CG31742	-	2L	0	*Prosβ5R1*	2R	1	0.3025	13.0583	0.0232	**1.05E-14**
Fad2	-	3L	0	*Desat1*	3R	4	0.2959	13.3822	0.0221	**2.92E-20**
CG6180	+	2L	0	*CG17919*	3R	1	0.4191	14.9749	0.0280	**9.78E-12**
CG9436	+	2R	0	*CG10638*	3L	3	0.323	15.9699	0.0202	**1.43E-25**
Arp53D	+	2R	0	*Actin88F*	3R	2	0.2478	16.2624	0.0152	**1.94E-36**
CG2577	+	X	0	*CkIα*	X	3	0.3118	19.5675	0.0159	**1.93E-31**
CG11825	+	2R	1	*CG17734*	3R	2	0.1141	0.9925	0.1150	**1.52E-06**
CG9920	+	3R	1	*CG11267*	3L	2	0.2549	1.805	0.1412	**5.21E-05**
Ef1α48D	+	2R	1	*Ef1α100E*	3R	4	0.0525	2.0441	0.0257	**1.09E-72**

Pp1-87B	+	3R	1	*Pp1α-96A*	3R	4	0.015	2.068	0.0072	**1.61E-68**
CanB	+	X	2	*CanB2*	2R	3	0.0094	4.4547	0.0021	**1.08E-42**
CG9602	+	3R	1	*Cr1*	X	3	0.4171	6.8996	0.0604	**4.26E-09**
CG9518	+	X	1	*CG9503*	X	2	0.3152	11.192	0.0282	**1.28E-43**
CG12169	+	3L	1	*CG17746*	3L	3	0.2909	12.2289	0.0238	**3.90E-18**
EfTuM	+	2R	1	*CG12736*	2R	2	0.4329	18.8044	0.0230	**3.60E-24**
CG6208	-	3R	0	*γSnap*	2R	6	0.1942	2.2169	0.0876	**5.71E-19**
CG14508	+	3R	1	*CG4769*	3L	5	0.2574	3.449	0.0746	**2.20E-21**
Gskt	+	3R	0	*Sgg*	X	9	0.3824	9.2092	0.0415	**4.09E-30**
CG11913	-	3R	1	*CG1970*	4	5	0.3059	11.9362	0.0256	**1.69E-36**
CG5078	-	3L	0	*CG11537*	3L	7	0.3417	13.1708	0.0259	**4.91E-25**
CG5718	+	3L	0	*Scs-fp*	2R	4	0.2783	14.4317	0.0193	**4.43E-44**
Vha16-2	-	3L	0	*Vha16*	2R	3	0.2297	7.5501	0.0304	**1.37E-21**
Vha16-3	+	3L	0	*Vha16*	2R	3	0.1078	7.6603	0.0141	**1.19E-26**
CG10748	+	3L	0	*CG7998*	3R	3	0.4389	12.0254	0.0365	**1.39E-17**
CG10749	+	3L	0	*CG7998*	3R	3	0.411	13.8675	0.0296	**1.52E-18**
CG18418	+	3L	0	*CG1907*	3R	1	0.3501	16.6715	0.0210	**1.54E-18**
CG7514	+	3L	0	*CG1907*	3R	1	0.3234	17.1302	0.0189	**6.87E-27**

Footnote: *Histone* and *amylase* genes were not included in our set. The reasons are that *histone* ancestral state is known to be intronless [14] and that the *amylase* parental gene is not present in the genome of *D. melanogaster* anymore [15].
