# Supplementary figures and images for: Comparative genomics reveals a constant rate of origination and convergent acquisition of functional retrogenes in Drosophila
Source: Genome Biol. 2007 Jan 18;8(1):R11. doi: 10.1186/gb-2007-8-1-r11 (PMC1839131; doi:10.1186/gb-2007-8-1-r11)

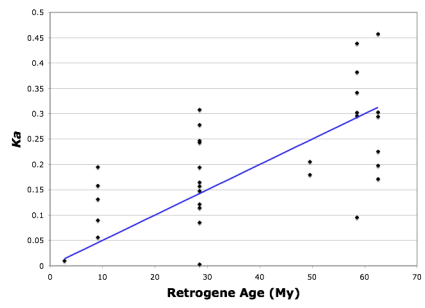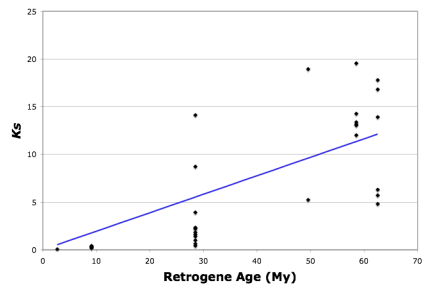

Supplement: Additional data file 3 — KS and KA correlation with our phylogenetic assignment (gene age estimate). [file gb-2007-8-1-r11-S3.pdf]

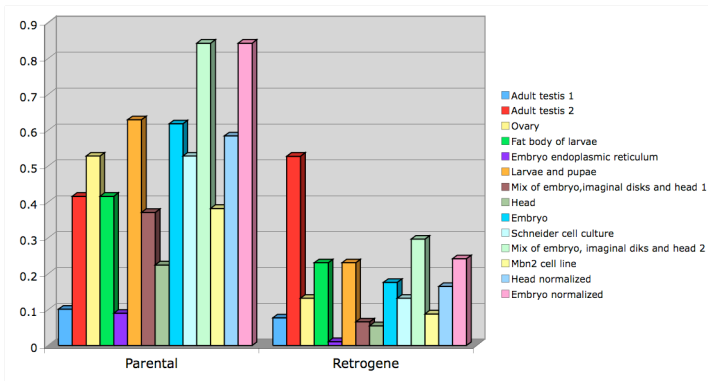

Supplement: Additional data file 5 — Proportions of parental genes and retrogenes expressed in every cDNA/EST library analyzed. [file gb-2007-8-1-r11-S5.pdf]
