# Supplementary material for: Comparative genomics reveals a constant rate of origination and convergent acquisition of functional retrogenes in Drosophila
Source: Genome Biol. 2007 Jan 18;8(1):R11. doi: 10.1186/gb-2007-8-1-r11 (PMC1839131; doi:10.1186/gb-2007-8-1-r11)
Supplement: Additional data file 4 — Statistical analysis of duplication between chromosomes. [file gb-2007-8-1-r11-S4.pdf]

Additional file 4. Analysis of duplication between chromosomes. Expected values were calculated following Betrán et al. (2002).

Direction	Expectation		Observation
	%	No	
X→A	23.3	13.5	30
A→X	20.3	11.8	10
A→A	56.4	32.7	18
X^2= 27.0496; df =2; P=0.000001			

X, X chromosome; A, Autosome.
