# Supplementary material for: Comparative genomics reveals a constant rate of origination and convergent acquisition of functional retrogenes in Drosophila
Source: Genome Biol. 2007 Jan 18;8(1):R11. doi: 10.1186/gb-2007-8-1-r11 (PMC1839131; doi:10.1186/gb-2007-8-1-r11)
Supplement: Additional data file 6 — A KS (Nei-Gojobori method) neighbor-joining tree of some members of the gene family is shown. Bootstrap values are shown in the nodes after 10,000 replications. MEGA [52] was used for this phylogenetic reconstruction. Chromosomal location was inferred from the location of flanking genes in D. melanogaster and is also given. [file gb-2007-8-1-r11-S6.pdf]

RET CG6036-RA PAR CG1906-RA

Ks tree

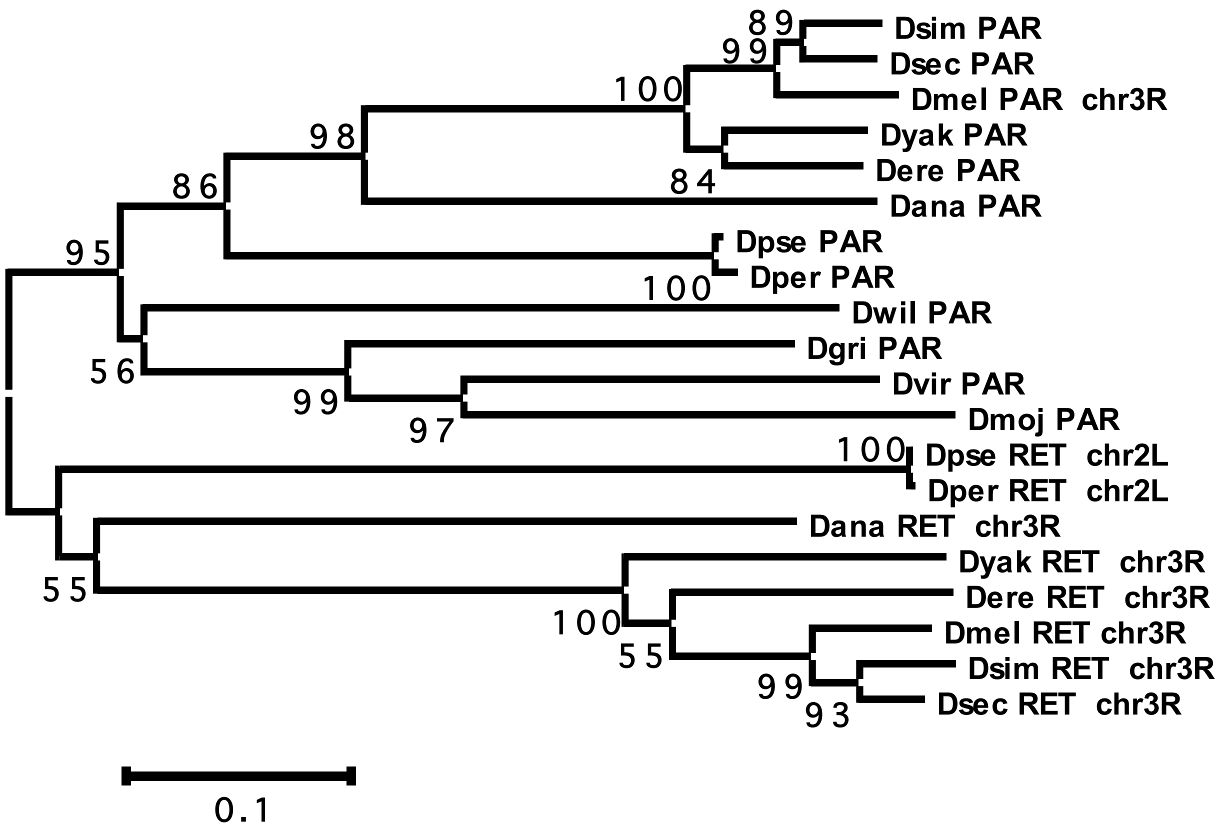

Protein tree

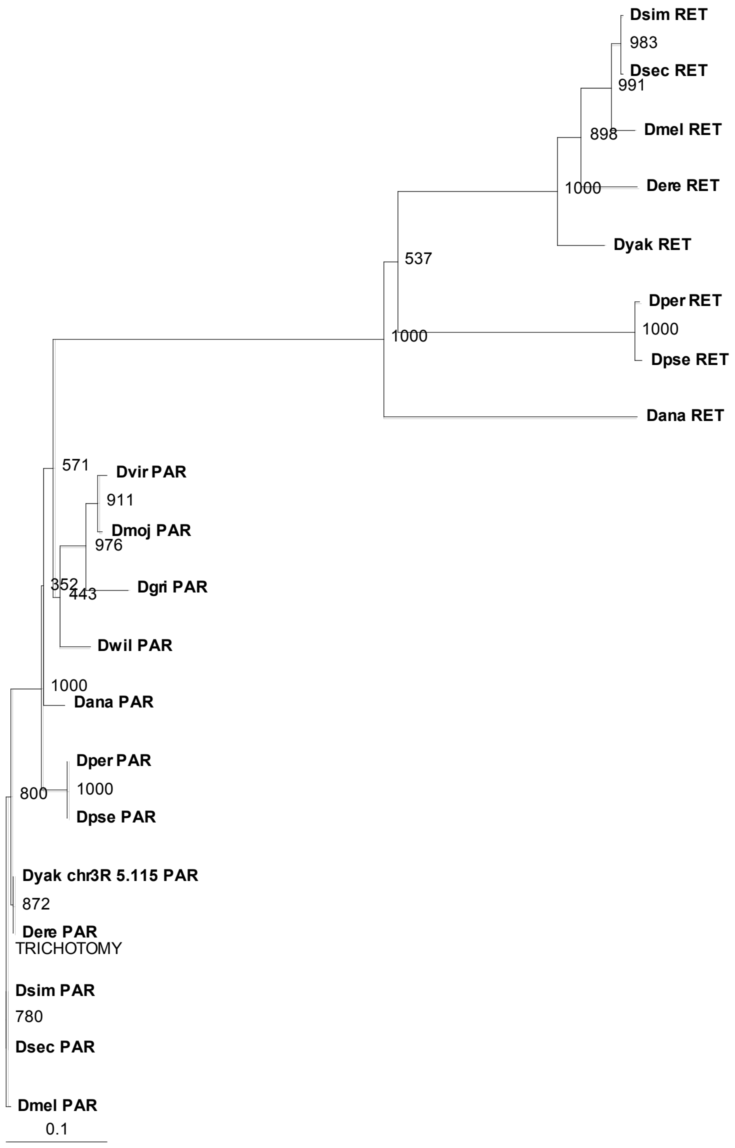

RET CG7423-RA PAR CG31715-RA

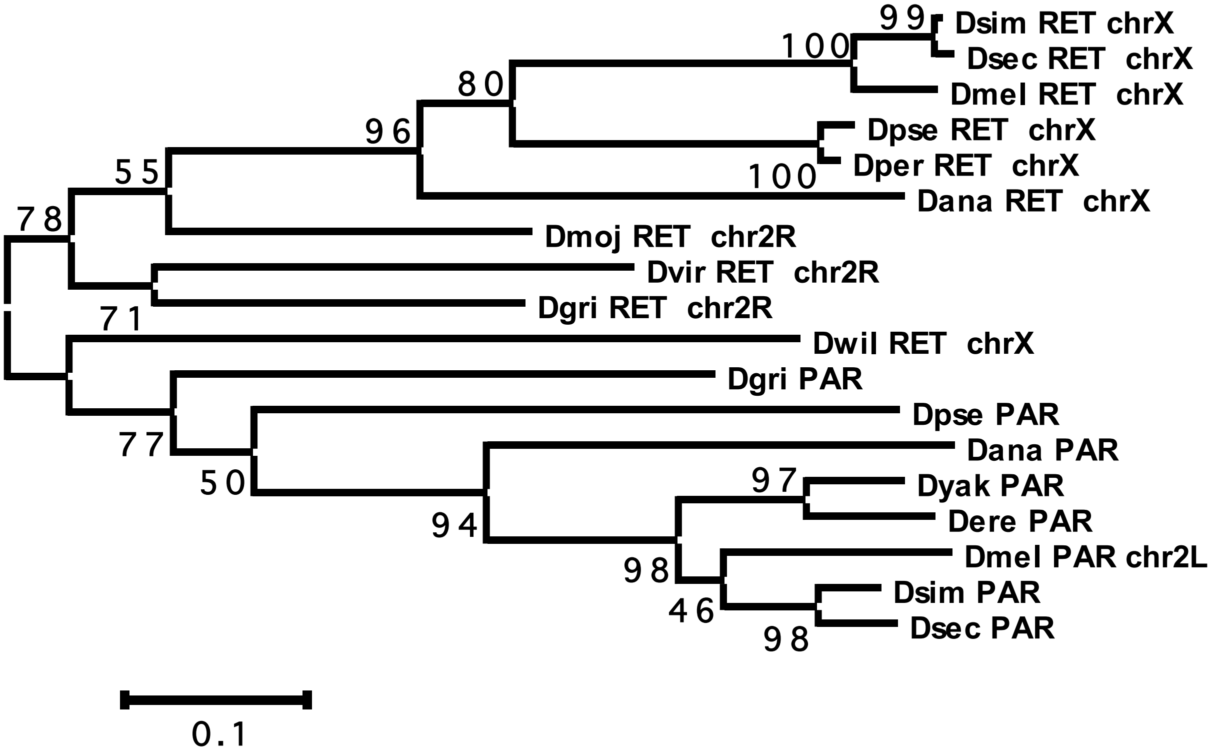
99
Dsim RET chrX
100
Dsec RET chrX
80
Dmel RET chrX
96
Dpse RET chrX
Dper RET chrX
55
100
Dana RET chrX
78
Dmoj RET chr2R
Dvir RET chr2R
Dgri RET chr2R
71
Dwil RET chrX
Dgri PAR
Dpse PAR
77
Dana PAR
50
97
Dyak PAR
94
Dere PAR
Dmel PAR chr2L
98
Dsim PAR
46
98
Dsec PAR
0.1

Protein

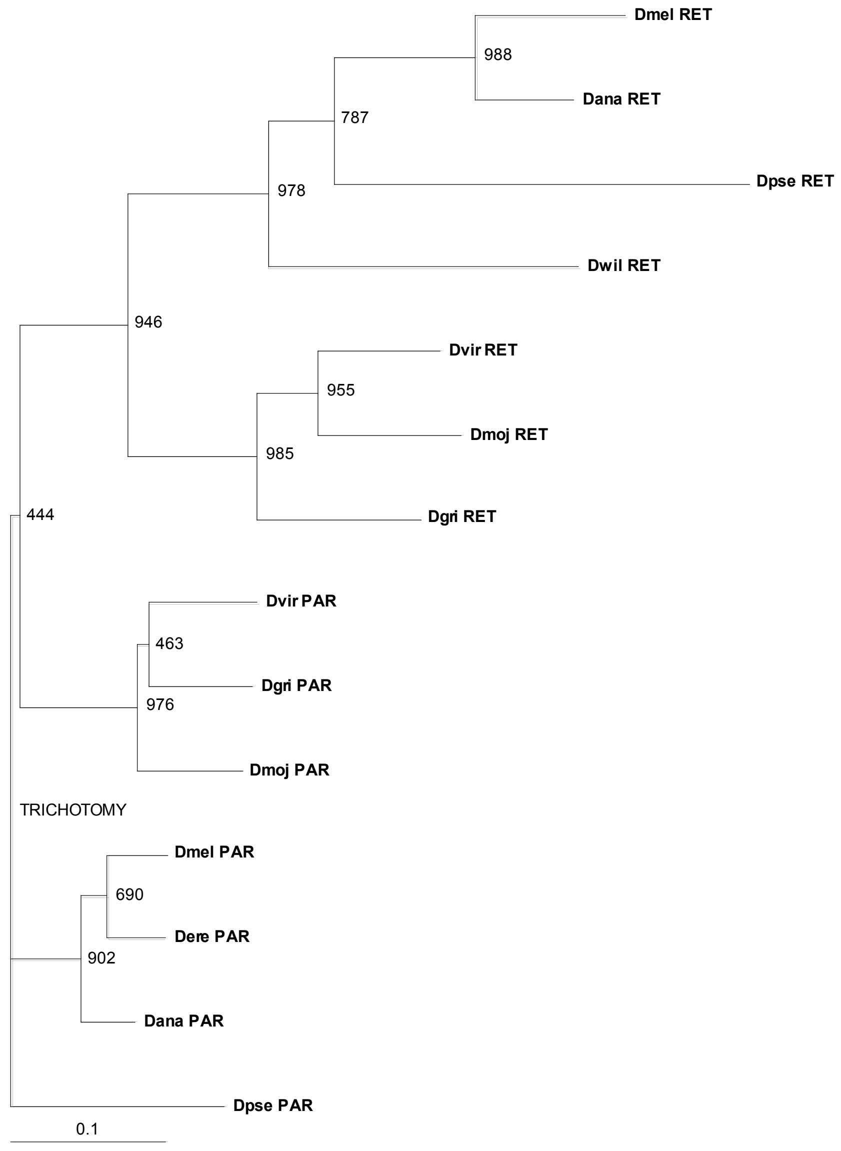

RET CG9013-RA 2R PAR CG3161-RB 2R

Ks tree

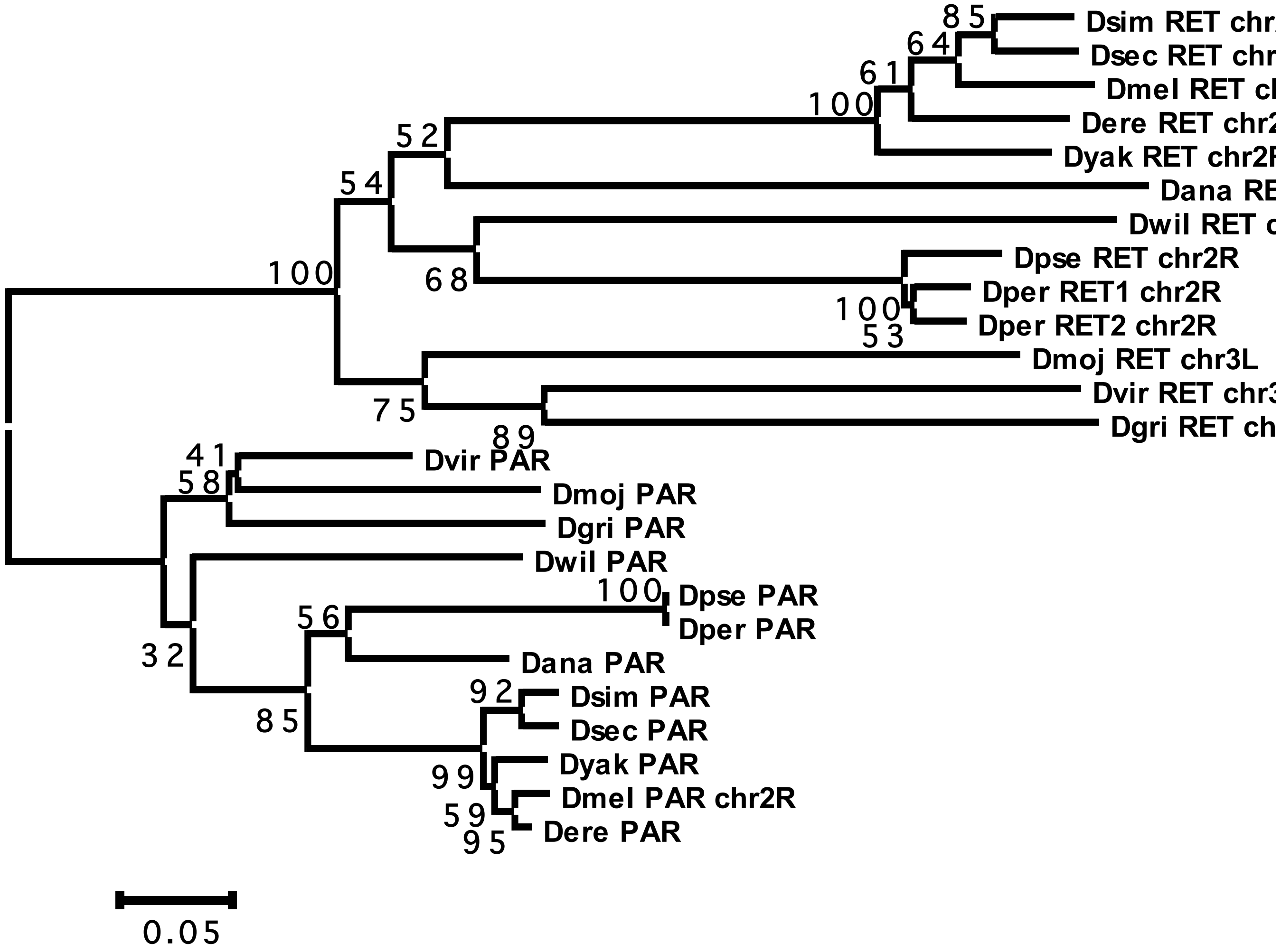
Dsim RET chr
Dsec RET chr
Dmel RET c
Dere RET chr2
Dyak RET chr2
Dana RE
Dwil RET c
Dpse RET chr2R
Dper RET1 chr2R
Dper RET2 chr2R
Dmoj RET chr3L
Dvir RET chr3
Dgri RET ch
Dvir PAR
Dmoj PAR
Dgri PAR
Dwil PAR
Dpse PAR
Dper PAR
Dana PAR
Dsim PAR
Dsec PAR
Dyak PAR
Dmel PAR chr2R
Dere PAR
85
64
61
100
52
54
100
68
100
53
75
89
41
58
32
100
56
85
92
99
59
95
0.05

Protein

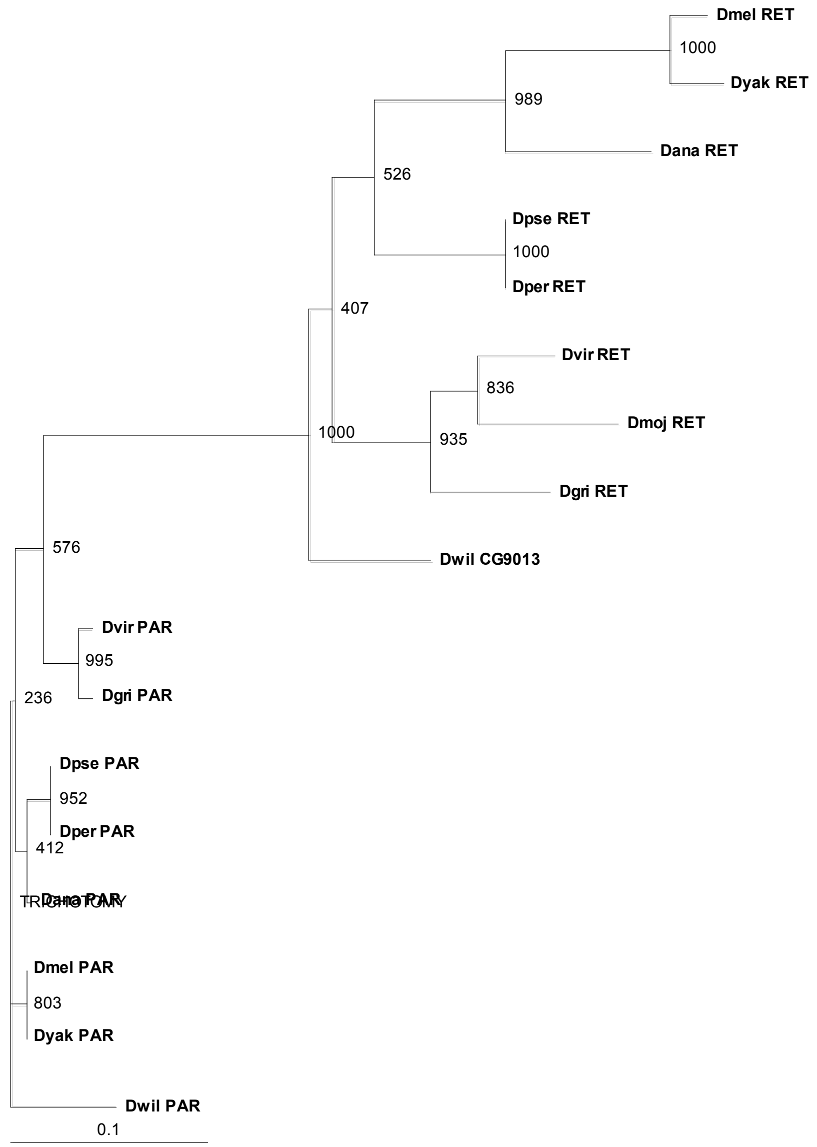

Dmel RET
1000
Dyak RET
989
Dana RET
526
Dpse RET
1000
Dper RET
407
Dvir RET
836
Dmoj RET
1000
935
Dgri RET
Dwil CG9013
576
Dvir PAR
995
Dgri PAR
236
Dpse PAR
952
Dper PAR
412
Dana PAR
TRICHOTOMY
Dmel PAR
803
Dyak PAR
Dwil PAR
0.1
