# Supplementary material for: Comparative genomics reveals a constant rate of origination and convergent acquisition of functional retrogenes in Drosophila
Source: Genome Biol. 2007 Jan 18;8(1):R11. doi: 10.1186/gb-2007-8-1-r11 (PMC1839131; doi:10.1186/gb-2007-8-1-r11)
Supplement: Additional data file 7 — A KA and KS (Nei-Gojobori method) neighbor-joining tree of some members of the gene family is shown. Bootstrap values are shown in the nodes after 10,000 replications. MEGA [52] was used for this phylogenetic reconstruction. Chromosomal location was inferred from the location of flanking genes in D. melanogaster and is also given. [file gb-2007-8-1-r11-S7.pdf]

RET CG13732-RA 3L PAR CG15645-RA X

Ks tree

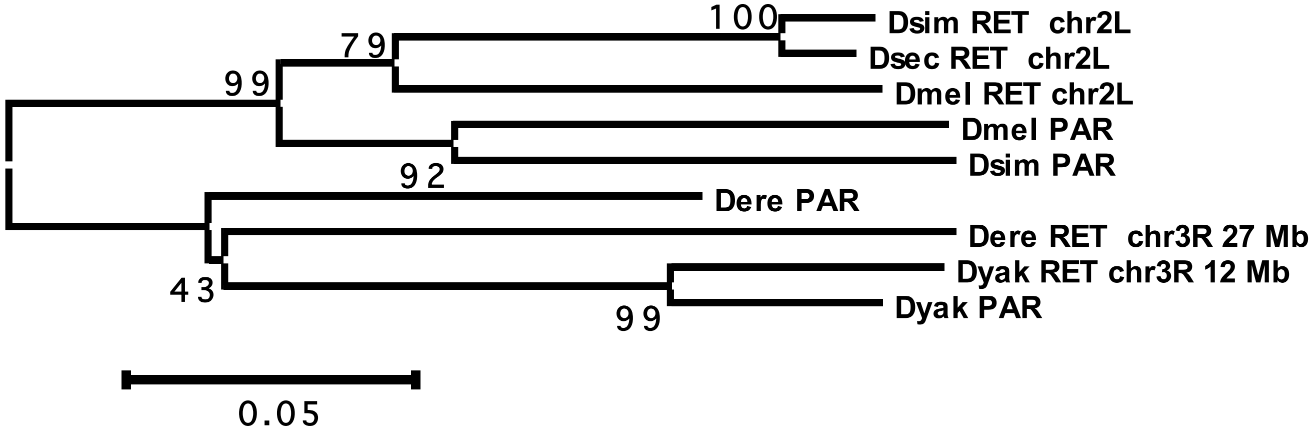

RET CG10174-RA 2L 9 PAR CG1740-RA X 1

Nuclear transport factor 2 (NTF2) domain. protein import into nucleus.

Ks tree

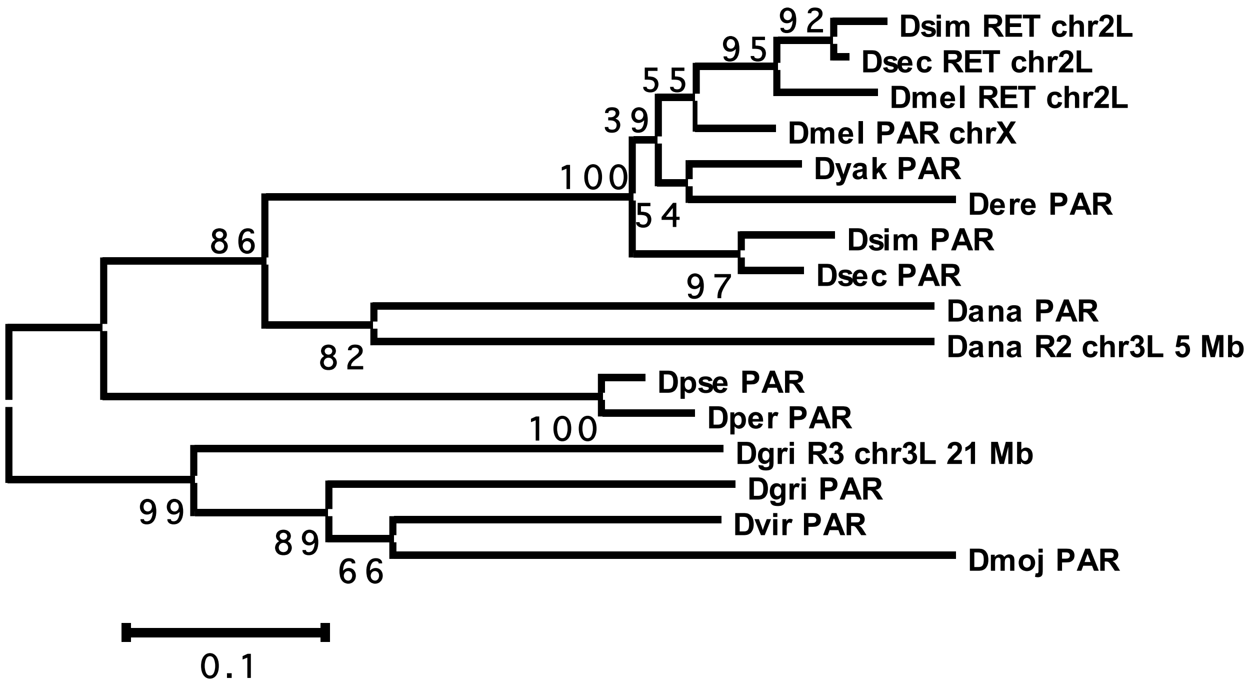

RET CG7815-RA 3L PAR CG1404-RB X

GTP-binding nuclear protein Ran-like. Belongs to the small GTPase superfamily. Ran family.

Ks tree

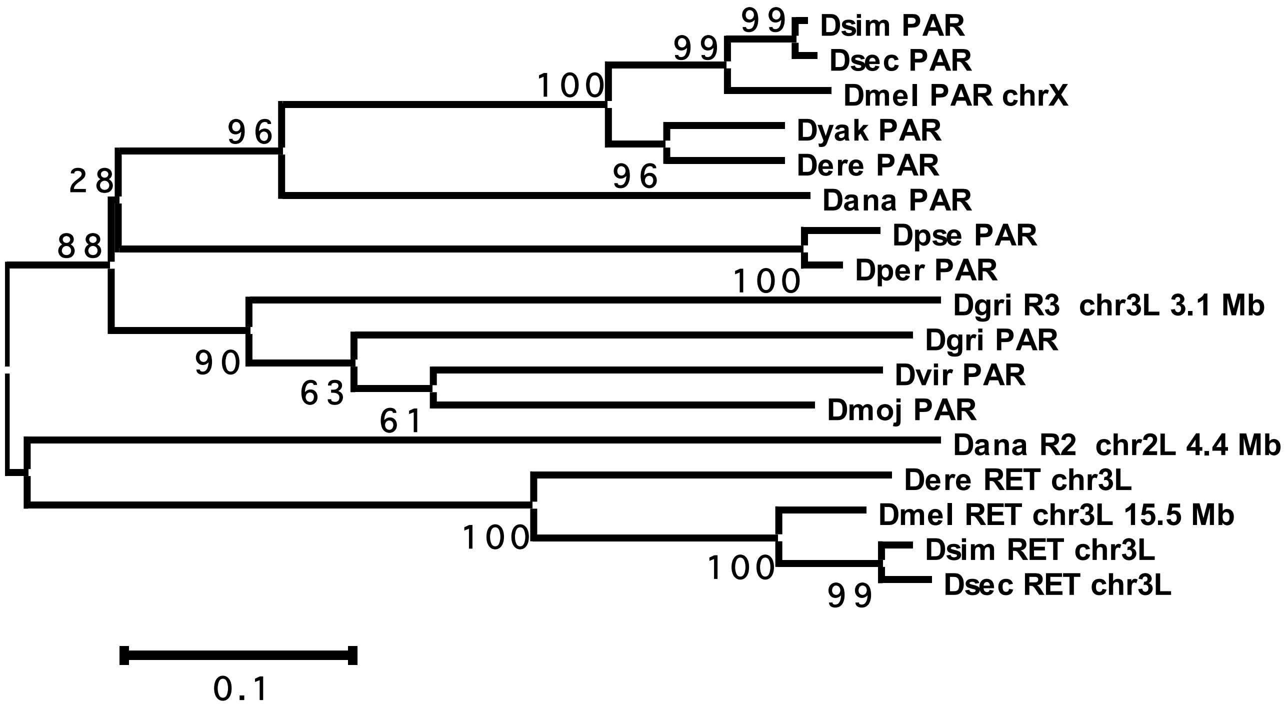

Footnote: Parental location is always conserved and is always X chromosome
